# Increased Rab35 expression is a potential biomarker and implicated in the pathogenesis of Parkinson's disease

**DOI:** 10.18632/oncotarget.11090

**Published:** 2016-08-05

**Authors:** Ching-Chi Chiu, Tu-Hsueh Yeh, Szu-Chia Lai, Yi-Hsin Weng, Yin-Cheng Huang, Yi-Chuan Cheng, Rou-Shayn Chen, Ying-Zu Huang, June Hung, Chiung-Chu Chen, Wey-Yil Lin, Hsiu-Chen Chang, Yu-Jie Chen, Chao-Lang Chen, Hsin-Yi Chen, Yan-Wei Lin, Yah-Huei Wu-Chou, Hung-Li Wang, Chin-Song Lu

**Affiliations:** ^1^ Neuroscience Research Center, Chang Gung Memorial Hospital at Linkou, Taoyuan, Taiwan; ^2^ Division of Movement Disorders, Department of Neurology, Chang Gung Memorial Hospital at Linkou, Taoyuan, Taiwan; ^3^ Department of Physiology and Pharmacology, Chang Gung University School of Medicine, Taoyuan, Taiwan; ^4^ Healthy Aging Research Center, Chang Gung University School of Medicine, Taoyuan, Taiwan; ^5^ College of Medicine, Chang Gung University, Taoyuan, Taiwan; ^6^ Graduate Institute of Biomedical Sciences, Chang Gung University School of Medicine, Taoyuan, Taiwan; ^7^ Department of Neurosurgery, Chiayi Chang Gung Memorial Hospital, Chiayi, Taiwan; ^8^ Institute of Cognitive Neuroscience, National Central University, Taoyuan, Taiwan; ^9^ Department of Nursing, Chang Gung University of Science and Technology, Taoyuan, Taiwan

**Keywords:** Parkinson's disease, Rab35, α-synuclein, biomarker, proteomics, Gerotarget

## Abstract

Parkinson's disease (PD) is the second common neurodegenerative disease. Identification of biomarkers for early diagnosis and prediction of disease progression is important. The present comparative proteomic study of serum samples using two-dimensional fluorescence differential gel electrophoresis followed by ELISA confirmation demonstrated that protein expression of Rab35 was increased in PD patients compared with matched control subjects and other parkinsonian disorders, progressive supranuclear palsy (PSP) and multiple system atrophy (MSA). The serum level of Rab35 was significantly correlated with the age at onset of PD. The median age of onset in patients with higher Rab35 serum level was 5 years younger than those with lower Rab35 serum level. There was a positive correlation between the Rab35 level and disease duration of PD. Moreover, the protein expression of Rab35 was increased in the substantia nigra but not in the striatum of mouse models of PD, including MPTP-treated mice, rotenone-treated mice, (R1441C) LRRK2 or (G2019S) LRRK2 transgenic mice. Furthermore, overexpression of Rab35 increased the aggregation and secretion of mutant A53T α-synuclein in dopaminergic SH-SY5Y cells. Co-expression of Rab35 with wild-type or A53T α-synuclein in SH-SY5Y cells deteriorated cell death. Our results suggest that Rab35 is potentially useful in the differential diagnosis of parkinsonian disorders and is implicated in the pathogenesis of PD.

## INTRODUCTION

Parkinson's disease (PD) is the second common age-related neurodegenerative disease with a prevalence of approximately 1-2% in people over 60 years of age [1]. The clinical presentations are evidently linked to the progressive degeneration of neuromelanin containing dopaminergic neurons in the pars compacta of the substantia nigra (SNpc) and the presence of Lewy bodies which are mainly composed of α-synuclein [2]. The etiology of PD is considered as an interaction of genes, environment, and aging process. Current established hypotheses concerning the pathogenesis of PD suggests the involvement of inflammatory process, increase in reactive oxygen species (ROS) and mitochondrial dysfunction, altered autophage-lysosome pathway, endoplasmic reticulum associated degradation, as well as ubiquitin-proteasome system (UPS) dysfunction, leading to abnormal protein folding and aggregation [3]. Up to now, the diagnosis of PD is based on the clinical observation but the accuracy for early diagnosis is limited owing to heterogeneous presentations. In addition, the treatment of PD is mainly symptomatic and there is no disease-modifying treatment that could halt or delay disease progression.

Blood-based biomarkers are widely reported in various diseases and identification of reliable biomarkers would be of great value in diagnosis of disease, prediction of progression, and development of therapy. Recent studies have discovered some protein biomarkers for the diagnosis of PD [4–13]; however, there is no recommended biomarker for routine clinical practice to date. The effectively clinical biomarker is urgently needed for early detection of PD and helps for management of PD.

In this study, we used proteomic approach to search for clinically useful serum biomarkers for PD. The two-dimensional gel electrophoresis based proteomic analysis identified that the serum level of a small GTPases Ras-related protein 35 (Rab35) was increased in PD patients. The elevated serum Rab35 protein is associated with younger onset of PD. Moreover, the increased expression of Rab35 was observed in MPTP-treated mice, rotenone-treated mice, (R1441C) LRRK2 or (G2019S) LRRK2 mice models for PD. Furthermore, overexpression of Rab35 promotes the aggregation and secretion of α-synuclein in SH-SY5Y cells. These results suggest that Rab35 is a potential biomarker for PD and implicated in the pathogenesis of PD.

## RESULTS

### Comparative serum proteomic analysis in PD patients

**Figure 1:**
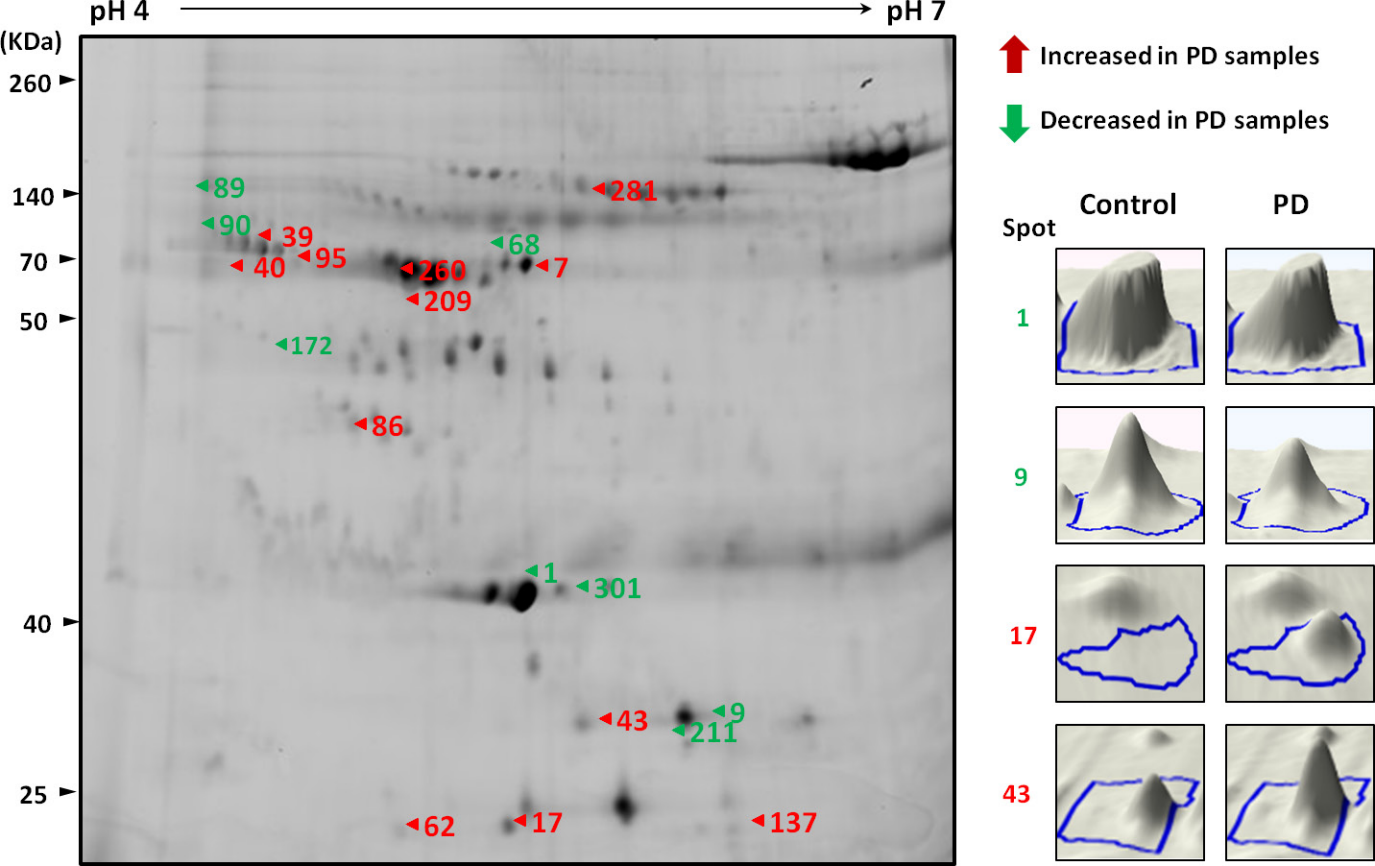
A representative composite two-dimensional difference gel electrophoresis of PD patients and healthy controls The arrows indicate the differentially expressed serum proteins in PD samples compared with healthy controls. The number in the gel corresponds to the master number presented in Table 1. The expression of proteins among serum samples from controls and PD patients were visualized by three-dimensional images (right panel).

The results of 2D-DIGE analysis of PD and NC serum pools revealed a differential expression pattern (Figure 1). The protein spots with at least 1.5-fold difference in abundance of the spot volume were selected for mass spectrometry (MS) identification. A total of 20 proteins were significantly differentially expressed (p < 0.05) in serum samples from PD compared to healthy controls, in which 11 proteins were overexpressed (average ratio > 1.5) while 9 proteins were downregulated (average ratio < −1.5) (Table 1).

**Table 1:** Relative changes in abundance of different proteins with at 1.5-fold between serum samples of PD patients and healthy controls

Spot no.^a^	Protein identification	GI Accession no.^b^	Mascot Score^c^	Coverage (%)^d^	Fold^e^	t- test^f^
1	Apolipoprotein A-IV	71773110	131	42	−2.86	0.0005
7	Serotransferrin	4557871	126	32	2.13	0.0031
9	Apolipoprotein A-I	4557321	149	64	−2.02	0.0002
17	Ras-related protein Rab-35	5803135	65	58	9.90	0.0017
39	Golgi integral membrane protein 4	7657138	61	13	1.54	0.0035
40	Protein disulfide-isomerase A4	4758304	68	40	1.77	0.0005
43	Solute Carrier Family 25, Member 5	46095315	56	45	1.97	0.0031
62	Transthyretin	4507725	95	73	2.52	0.0010
68	Ceruloplasmin	4557485	120	27	−2.14	0.0008
86	Trans-Golgi network integral membrane protein 2	332205949	57	11	3.63	0.0012
89	Complement C3	115298678	94	16	−1.76	0.0006
90	Epidermal growth factor receptor substrate 15	4503593	62	23	−1.79	0.0002
95	Prolyl endopeptidase-like	153217451	57	36	1.64	0.0088
137	Apolipoprotein A-II	4502149	74	87	1.99	0.0003
172	26S protease regulatory subunit 7	547930	61	69	−3.13	0.0003
209	Protein unc-45 homolog A	74761419	64	11	1.63	0.0057
211	Ras-related protein Rab-39	39930371	62	50	−1.75	0.0070
260	Vitamin D-binding protein	324021743	70	29	1.89	0.0001
281	Alpha-2-macroglobulin	66932947	68	31	−1.73	0.0000
301	Haptoglobin	386783	65	27	−1.64	0.0029

a Refers to the master spot number on the 2D-DIGE generated by DeCyder image analysis software.

b Accession number from NCBInr database.

c Score was calculated by Mascot search engine and related to the probability assignment.

d Coverage indicates the ratio of matched peptides in the protein sequence.

e Fold in ratio of the protein abundance between PD and control group.

f Independent tests between PD and healthy control group.

### Validation of differentially expressed Rab35 protein in serum samples

**Figure 2:**
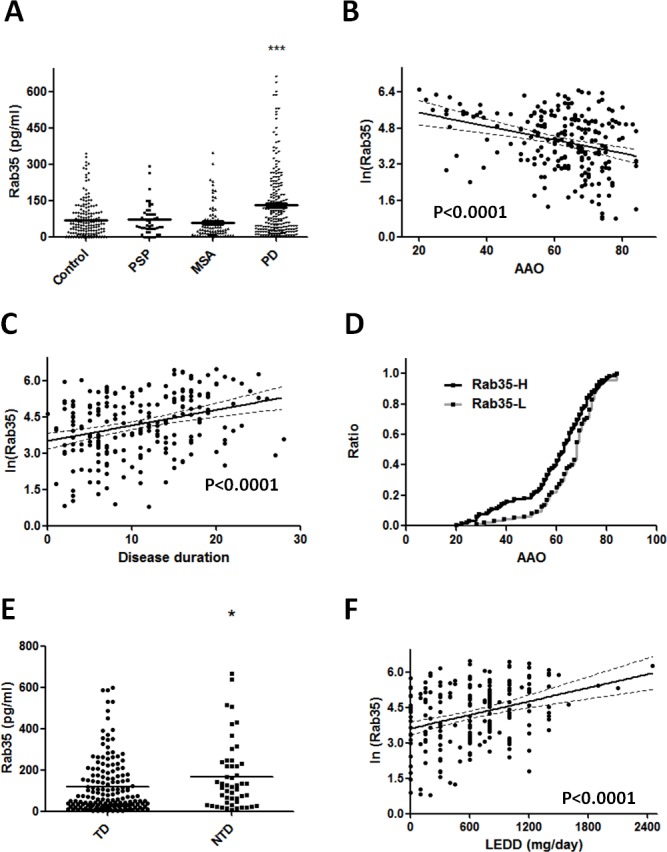
Rab35 is elevated in serum samples from patients with PD (**A**) The dot plot shows serum concentrations of Rab35 in healthy control subjects (n=177), PSP patients (n=46), MSA patients (n=80) and PD patients (n=213). ***p < 0.001 compared to control, MSA or PSP group. (**B**) The linear regression analysis shows a good correlation between Rab35 concentration and age at onset (AAO) with fitted regression line (p < 0.0001) and the value of Rab35 increased by 0.0184 when AAO decreased one unit (year). (**C**) There is also a good correlation between expression of Rab35 and disease duration in PD patients (p < 0.0001). (**D**) The onset age (AAO) of PD patients with high protein level of Rab35 (Rab35-H, n=119) was significantly younger than that of those with low protein level of Rab35 (Rab35-L, n=94) (difference of median age of onset = 5 years, p = 0.019, log-rank test). (**E**) The serum level of Rab35 protein was lower in tremor-dominant (TD) PD patients than in those non-tremor-dominant (NTD) patients with predominant akinetic-rigid syndrome (119.68±10.31 vs. 167.71±23.22 pg/ml, p=0.032). (**F**) Linear regression analysis showed the positive correlation between LEDD and the serum level of Rab35.

Among the differentially expressed serum proteins, Rab35 has 9.9-fold change in the abundance of serum protein in PD patients compared with controls. To validate the differential expression of Rab35 protein in serum samples, 213 PD patients, 46 PSP patients, 80 MSA patients, and 177 healthy control subjects were included for quantitative ELISA analysis. We choose PSP and MSA cases as disease controls because these parkinsionian disorders are clinically indistinguishable to PD at early stage and pathologically distinct. The mean serum concentrations of Rab35 in healthy controls, PSP, MSA and PD patients were 70.51±5.72, 71.22±9.37, 58.37±8.22 and 131.30±9.67 pg/ml, respectively (Supplemental Table 1 & Figure 2A). The ANOVA analysis (F=15.18, p < 0.0001) followed by post-hoc multiple comparison using Tukey method showed that the level of Rab35 was significantly higher in serum samples of PD patients compared with control subjects (1.86-fold change, p < 0.0001), PSP group (1.84-fold change, p < 0.01) and MSA group (2.25-fold change, p < 0.0001). Moreover, we carried out a linear regression analysis to examine the association of serum level of Rab35 protein with the clinical parameters. A significant correlation was observed between the serum concentration of Rab35 (Y, pg/ml) and age at onset of PD (X, years) (Y = ln(−0.02996*X + 6.085), p < 0.0001) (Figure 2B). The serum level of Rab35 was also correlated with the disease duration (X, years) of PD (Y = ln(0.07050*X + 3.437), p < 0.0001) (Figure 2C). Additionally, we compared the age at onset (AAO) of PD with serum Rab35 above or below the calculated cut-off value (60 pg/ml) by using log-rank (Mantel-Cox) test (Figure 2D). PD patients with higher level of Rab35 (> 60 pg/ml; n=119) in sera had a younger onset age of disease compared to patients with lower expression of Rab35 (n=94) (difference of median age of onset = 5 years, p = 0.019). The analysis of clinical phenotype revealed that tremor-dominant PD patients had lower Rab35 serum level than those patients with predominant akinetic-rigid syndrome (119.68±10.31 vs. 167.71±23.22 pg/ml, p=0.032) (Figure 2E). There was no difference of Rab35 expression between familial and sporadic cases. In addition, there was a significant correlation between treatment dose (levodopa equivalent daily dose, LEDD) and the protein level of Rab35 (Figure 2F, p<0.001). To further investigate the characteristics of PD patients with a high level of Rab35 (Rab35 level >190 pg/ml in 25% of patients), we analyzed tremor-dominant phenotype, the levodopa equivalent daily dose, disease duration and onset age of disease in PD patients with a high level of Rab35. This group of patients has significantly younger onset age (52.45±2.33 vs. 64.94±0.88 years, p<0.001), longer disease duration (19.58±1.05 vs. 12.84±0.61 years, p<0.001), larger LEDD (893.75±66.64 vs. 611.92±28.6 mg, p<0.001), and less tremor-dominant phenotype (69% vs 79%).

**Figure 3:**
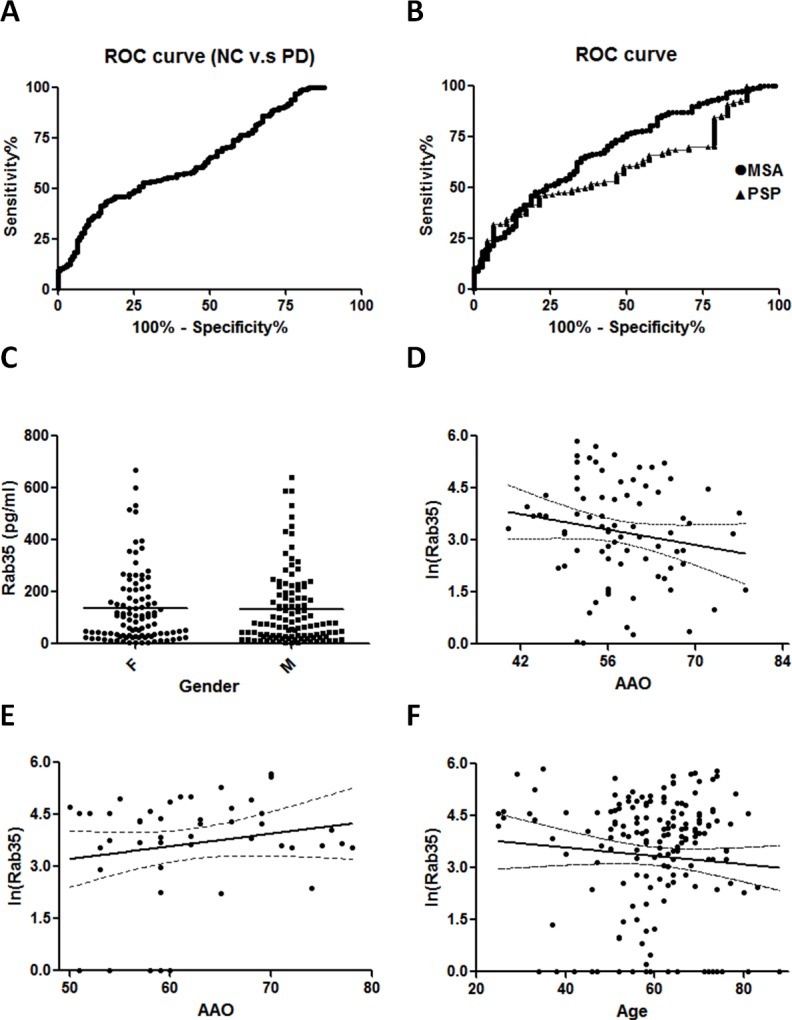
Correlation analysis of gender, age, age of onset and serum level of Rab35 (**A**, **B**) Receiver operating characteristic (ROC) curves of Rab35 serum level. The ROC area under curve (AUC) for PD were 0.664 (95% CI, 0.605-0.722) vs. NC, 0.601 (95% CI, 0.522-0.680) vs. PSP and 0.690 (95% CI, 0.623-0.756) vs. MSA. 95% CI: 95% confidence interval. (**C**) Distribution of the level of Rab35 in male and female PD groups. The expression of Rab35 was measured by ELISA assay. There were no significant differences in gender between the PD group (F=106, M=107, p=0.7775). (**D**-**F**) Correlation analysis of serum Rab35 levels in (**D**) MSA (n=80, p=0.1686), (**E**) PSP (n=47, p=0.2085) and (F) healthy control subjects (n=177, p=0.2678). Abbreviation: F, female; M: male.

The diagnostic value of Rab35 serum level was assessed by using Receiver Operating Characteristic (ROC) curves. The ROC areas under curves (AUC) for PD vs. NC, PSP, and MSA were 0.6351 (95% CI, 0.5805-0.6898), 0.6029 (95% CI, 0.5238-0.6820), and 0.6914 (95% CI, 0.6248-0.7579), respectively (Figure 3A and B). While the cut-off value of Rab35 was set as 60 pg/ml, the sensitivity was 56.34% and the specificity for PD vs NC, PSP, and MSA were 55.93%, 53.19%, and 68.75%, respectively. There is a better discrimination between PD and MSA cases. There was no gender difference of Rab35 serum level in PD patients (Figure 3C). The Rab35 serum levels were not correlated with the age of control groups or AAO of PSP and MSA patients (Figure 3D-F). To demonstrate that an increase of Rab35 levels is a specific feature of PD, we examined the serum level of Rab35 in patients affected with non-parkinsonism CNS diseases, including Alzheimer's disease (AD), spinocerebellar ataxia (SCA) and Huntington's disease (HD). An increased serum level of Rab35 was not observed in AD, SCA or HD patients (Supplemental Figure 2). These data indicate that the increased expression of Rab35 might represent a biomarker to differentiate PD patients from other parkinsonian disorders and is correlated with younger onset age of PD.

### Expression of Rab35 in MPTP-treated mice, rotenone-treated mice, (R1441C) LRRK2 or (G2019S) LRRK2 transgenic mice

**Figure 4:**
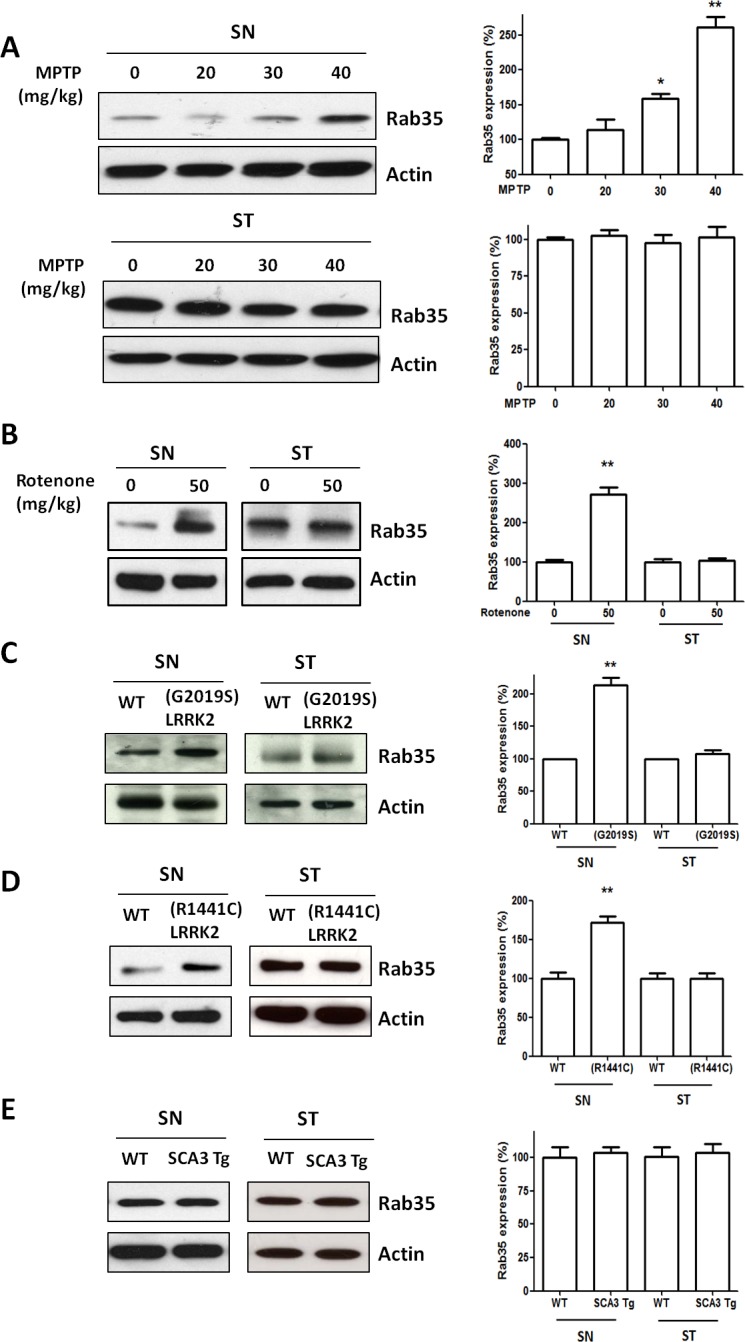
The protein level of Rab35 is increased in the substantia nigra (SN) of MPTP-treated mice, rotenone-treated mice, (G2019S) LRRK2 or (R1441C) LRRK2 transgenic mice, but not in the SN of SCA3 transgenic mice (**A**) Treatment with MPTP significantly increased protein level of Rab35 in the SN. Quantification of Rab35 protein measured by the use of a densitometer showed that MPTP administration induced an increase in protein level of Rab35 in the SN with a dose-dependent manner. (**B**) The protein level of Rab35 was increased in the SN of rotenone-treated mice. (**C**) In contrast to striatum (ST), the expression of Rab35 protein in the SN was significantly upregulated in (G2019S) LRRK2 transgenic mouse. (**D**) The protein level of Rab35 in the SN was increased in (R1441C) LRRK2 transgenic mouse. (**E**) There was no significant difference in protein expression of Rab35 between wild-type mouse and SCA3 transgenic mouse. Each bar represents the mean ± SEM value of four independent experiments. *p < 0.05, **p < 0.01 compared to control groups.

To study whether the increased serum Rab35 level reflects the alteration of Rab35 protein in the brain, we measured the Rab35 protein expression in the substantia nigra (SN) and striatum (ST) in non-genetic PD mouse models (MPTP- or rotenone-treated mouse) and genetic PD mouse models ((R1441C) or (G2019S) LRRK2 transgenic mouse). MPTP and rotenone treatment induces mitochondrial dysfunction and causes cell death of SNpc dopaminergic neurons. Two weeks after the administration of MPTP or rotenone, we observed an increase of Rab35 protein level in the SN but not in the ST of MPTP- or rotenone-treated mouse (Figure 4A and 4B).

We previously generated the transgenic mouse model of mutant LRRK2-induced PD. (R1441C) LRRK2 or (G2019S) LRRK2 transgenic mouse at the age of 12-16 months displayed parkinsonism phenotypes of motor dysfunction and cell death of SNpc dopaminergic neurons [14,15]. Compared with wild-type mice, protein expression of Rab35 was significantly increased in the SN of 12-month-old (G2019S) LRRK2 transgenic mice (Figure 4C) or 16-month-old (R1441C) LRRK2 transgenic mice (Figure 4D). In contrast, protein level of Rab35 was not altered in the ST of (G2019S) LRRK2 or (R1441C) LRRK2 transgenic mice (Figure 4C and 4D). Moreover, we also examined the protein expression of Rab35 in the transgenic mouse model of SCA3 [16]. The protein level of Rab35 in the SN of SCA3 transgenic mouse was similar to that of wild-type mouse (Figure 4E). Region-specific increased expression of Rab35 protein in the SN of MPTP-treated mouse, rotenone-treated mouse, (R1441C) LRRK2 or (G2019S) LRRK2 transgenic mouse suggests that Rab35 may play an important role in the pathogenic mechanism of PD.

### Rab35 promotes aggregation and secretion of A53T α-synuclein

**Figure 5:**
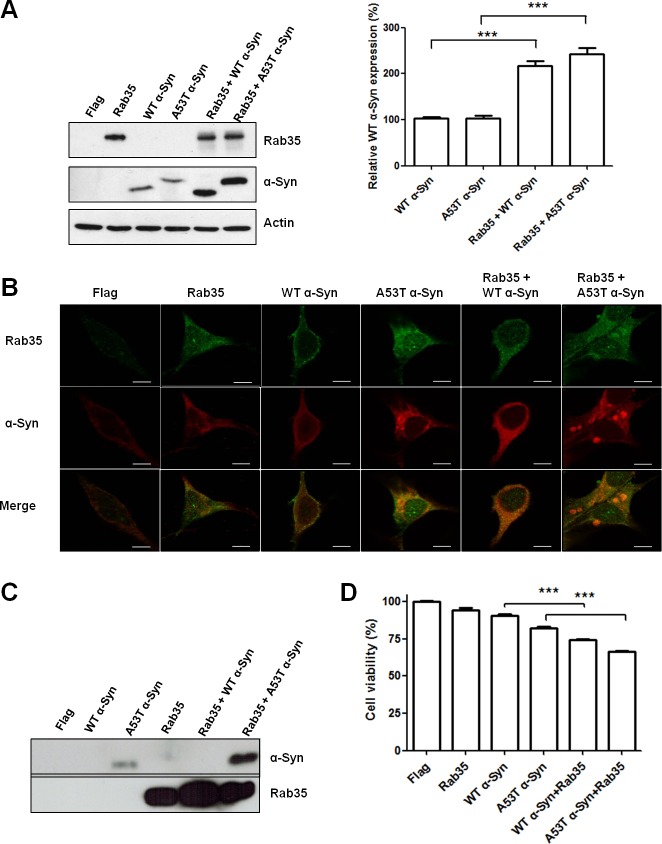
Rab35 promotes aggregation and secretion of A53T α-synuclein (A53T α-Syn) (**A**) The overexpression of Rab35 significantly increased the protein expression of wild-type α-Syn and mutant A53T α-Syn in SH-SY5Y cells (**B**) The immunofluorescent staining showed that overexpression of Rab35 increased the aggregation of A53T α-Syn in SH-SY5Y cells. (anti-Rab35 antibody – green, anti-α-synuclein antibody – red; Scale bar is 10 μm) (**C**) After 48 hours transfection, SH-SY5Y were grown in the cell culture medium containing 2% FBS. The conditioned medium (CM) was collected. The immunoblotting assay demonstrated that Rab35 overexpression enhanced the release of A53T α-Syn into CM. (**D**) Overexpression of Rab35 increases cell death in WT α-Syn and A53T α-Syn transfected SH-SY5Y cells. After 48 hours of transfection, Cell Counting Kit (CCK)-8 was used to analyze cell survival of transfected cells. Data in graphs represent the mean ± SEM of four independent experiments. ***p < 0.001 compared to WT α-Syn or A53T α-Syn-transfected cells.

Aggregation of α-synuclein (α-Syn) has been regarded as the histological hallmark of PD [17,18]. Strong association of SNCA gene in sporadic PD has been observed in genome-wide association studies (GWAS) [19]. Recent studies also showed that α-Syn interacts with several members of the Rab family [20–22]. To investigate whether Rab35 is involved in the aggregation and secretion of α-synuclein, we expressed wild-type (WT) α-Syn, A53T α-Syn, Rab35, WT α-Syn plus Rab35 or A53T α-Syn plus Rab35 in SH-SY5Y cells. The overexpression of Rab35 significantly increased the protein expression of wild-type α-Syn and mutant A53T α-Syn in SH-SY5Y cells (Figure 5A). Next, we investigated whether expression of Rab35 could promote the formation of α-Syn aggregates by using confocal microscopic imaging analysis. The immunocytochemical staining using anti- α-synuclein and Rab35 antibodies demonstrated the formation of α-synuclein aggregates in SH-SY5Y cells expressing A53T α-Syn but not in cells transfected with WT α-Syn or Rab35. The presence of Rab35 caused a significant increase in the formation of A53T α-Syn aggregates in SH-SY5Y cells (Figure 5B). The α-synuclein has been detected in biological fluids, such as blood plasma and cerebrospinal fluid (CSF). It could be secreted in the culture medium of neuronal cells. The mechanism of α-synuclein release is not clear, but secretory pathway that involves vesicle trafficking has been proposed [23]. To examine the effect of Rab35 on secretion of α-synuclein, conditioned medium (CM) was collected 48 hours after transfection and the presence of Rab35 and α-Syn in the CM were detected by using immunoblotting assay. As expected, the secretion of A53T α-Syn was detectable in the CM from mutant A53T α-Syn-expressing SH-SY5Y cells (Figure 5C). Co-transfection of Rab35 with mutant A53T α-Syn significantly increased the secretion of A53T α-Syn in the CM (11.63-fold, p = 0.0026). There was no significant change of lactate dehydrogenase (LDH), an indicator of cell membrane integrity, in the CM (data not shown). Furthermore, cell survival was assessed by WST-8 [2-(2-methoxy-4-nitrophenyl)-3-(4-nitrophenyl)-5-(2,4-disulfophenyl)-2H-tetrazolium, monosodium salt] assays using Cell Counting Kit-8. Co-expression of Rab35 with either WT α-Syn or A53T α-Syn in SH-SY5Y cells resulted in decreased cell viability (Figure 5D). Collectively, the results of enhancement of the aggregation and secretion of mutant A53T α-Syn by Rab35 suggest that Rab35 is involved in the pathogenesis in PD.

## DISCUSSION

In this study, we used 2D-DIGE to analyze the serum proteomic profiles of PD and identified 20 differentially expressed proteins in the serum from PD patients (Supplemental Table 2). Some of them have been found in previous studies [24–27]. Among these proteins, Rab35 protein was markedly increased in PD patients. The increased serum level of Rab35 was confirmed by ELISA analysis. Interestingly, the expression level of Rab35 was not altered in PSP or MSA patients. It suggests that the change of Rab35 protein is specific for PD and there are different pathogenic mechanisms involved in these parkinsonian disorders.

The mammalian Rab GTPases are evolutionarily conserved, and there are more than 60 Rabs identified in human. Rabs are molecular switches, activated by guanine-nucleotide exchange factors (GEFs) and inactivated through GTPase-activating proteins (GAPs), regulate many steps of membrane traffic and recycling, including vesicle formation, vesicle movement along actin and tubulin networks, membrane fusion as well as intracellular signaling in a temporally and spatially sensitive manner [28, 29]. Rab35 is phylogenetically conserved in metazoans and is localized to the plasma membrane, clathrin-coated pits, vesicles and endosomal membranes. It has been shown to regulate the endocytic recycling of numerous protein cargo, the recycling of synaptic vesicles, the fusion of exosomes and the actin cytoskeleton [28, 30, 31]. Recent studies suggest that Rab35 is a key regulator in nerve growth factor (NGF)-induced neurite outgrowth. Rab35, together with its effectors MICAL-L1 and centaurin-β2, recruits EHD1 (a facilitator of vesicle formation) to Arf6-positive perinuclear recycling endosomes in response to NGF stimulation [32, 33]. In addition, Rab35 is involved in oligodendrocyte differentiation and myelination. Rab35 and another effector, ACAP2, a GTPase-activating protein that switches off Arf6 activity, negatively regulate oligodendrocyte morphological differentiation [34]. These studies indicate that Rab35 actively participates in many functions in the nervous system. Implication of Rab35 in PD pathogenesis has not been reported but recent studies suggest the possible link between the Rab protein family and the pathogenesis of PD. The genetic analysis identified loss of function mutation in Rab39B gene in two families with early-onset parkinsonism and intellectual disability and loss of Rab39B expression was observed in the postmortem brain from PD patients [35]. Patients with a truncating mutation (p.Trp186stop mutation) of Rab39B display typical parkinsonism and early disease onset [36]. A recent study suggested that G192R mutation of Rab39B possibly resulted in mislocalization and altered its intracellular targeting [37]. RAB7L1, located in the PARK16 locus identified from GWAS, has been shown to interact with LRRK2 to modify intracelluar protein sorting and risk for PD [38]. Moreover, recent study in Drosophila model demonstrated that Rab11 decreases α-synuclein aggregation and ameliorates α-synuclein dependent phenotypes including locomotor activity, degeneration of dopaminergic neurons and shortened lifespan [39]. Therefore, we hypothesized that Rab35 plays an important role on the pathogenesis in PD (Supplemental Figure 1). In normal condition, Rab35 is localized in recycling endosome trafficking to the cell surface. In PD model, the dysfunction of protein degradation could result in the sequestration of abnormal proteins in Rab35 localized endosome. The present study showed that overexpression of Rab35 promotes the aggregation of mutant A53T α-Syn and secretion of mutant A53T α-Syn to the intercellular space. The results suggest that Rab35 may actively participate the processing and trafficking of α-synuclein and that endocyclic recycling may be involved in the pathogenesis of PD. Further studies are required to elucidate the regulatory mechanism of Rab35 expression in PD.

There are some limitations in this study. One is a smaller sample size for PSP and MSA due to the relatively lower incidence than PD. The other is all samples from a single center. Nevertheless, the findings on the animal models support the observation from human samples. Searching for collaboration to enroll samples from multiple centers will further validate the results. Another unanswered question is what mechanism is involved to increase Rab35 expression in PD. Is this a signal of neuronal injury or death rather than an accomplice? Further study is required to address these issues.

The present study shows that the serum level of Rab35 is elevated in PD patients but not in PSP or MSA patients and is negatively correlated with the onset age of PD. Increased Rab35 expression is also observed in SN tissue from PD mouse models including MPTP-treated mouse, rotenone-treated mouse, (R1441C) LRRK2 or (G2019S) LRRK2 transgenic mouse. In addition, Rab35 promotes the aggregation and secretion of A53T α-synuclein. These results suggest that Rab35 may be a biomarker for PD and is implicated in the pathogenesis of PD.

## MATERIALS AND METHODS

### Studying subjects

This study was approved by Institution Review Board of the hospital (IRB no. 102-0949A3). A written informed consent was obtained from all participants. We enrolled 213 patients with sporadic idiopathic PD, 46 patients with progressive supranuclear palsy (PSP), 80 patients with multiple system atrophy (MSA) and 177 healthy ethnically matched control subjects. The clinical diagnoses of PD, PSP and MSA, AD were based on the United Kingdom PD Brain Bank criteria [40], National Institute for Neurological Disorders and Society for PSP (NINDS-SPSP) [41], and the consensus statement on the diagnosis of multiple system atrophy [42], respectively. All the participants were interviewed by one of the authors. The demographic data were shown in Supplemental Table 1.

### Collection of serum samples

Serum samples were obtained from enrolled subjects and processed within 2 hours. Peripheral whole blood samples were collected in 5 ml BD Vacutainer glass tubes without additive (BD Biosciences) and allowed to clot at room temperature for 30 min. Serum was separated by centrifugation at 3000 rpm for 15 min at 4°C. The samples were then divided into aliquots and stored at −80°C.

### Proteomic analysis on two-dimensional difference gel electrophoresis (2D-DIGE)

The present study used two-dimensional difference gel electrophoresis (2D-DIGE)-based proteomic analysis of serum proteins to identify the differentially expressed proteins between PD patients and healthy control subjects (NC) (the samples were pooled from n=25 for each group). Albumin and IgG were removed from serum samples using a ProteoExtract Albumin/IgG Depletion Kit (Sigma-Aldrich). The depleted serum samples were labeled with CyDye DIGE Fluors according to the manufacturer's instructions (GE Healthcare). In brief, 50 μg depleted serum from PD patients and control individuals were labeled with Cy3 and Cy5, respectively. For internal standard, equal amounts of the samples was labeled with Cy2. Samples were then applied to immobilized pH gradient (IPG) strips (18 cm, pH 4-7) (GE Healthcare) for 12 h rehydration. Isoelectric focusing (IEF) was conducted using an Ettan IPGphor 3 IEF system (GE Healthcare) at 250 V for 20 min, gradually increased to 10,000 V with 3 h, and held at 10,000 V for a total 50,000 Voltage hours (Vh) at 20°C. Following IEF, strips were equilibrated and then transferred onto 12% SDS-PAGE and sealed with 1% agarose. The second dimension separation was performed on an Ettan Dalt-6 electrophoresis system (GE Healthcare). The images were analyzed using DeCyder V5.0 software (GE Healthcare). Spots displaying a ≥ 1.5 average-fold change in abundance with p values < 0.05 (Student's t-test) were marked and selected for protein identification. Protein identification by peptide mass fingerprints (PMFs) and MS/MS spectra was performed using the MASCOT search engine 2.2 (Matrix Science Inc., MA, USA) and the BioTools software version 3.0 (Bruker Daltonics) against the SwissProt or NCBInr databases.

### Quantification of Rab35 serum level by enzyme linked immunosorbent assay (ELISA)

Serum concentrations of Rab35 were measured by using Human Ras-related protein Rab-35(RAB35) ELISA kit according to the manufacturer's instructions (Cusabio, Wuhan, China). In brief, 100 μl of serum samples from patients and controls were added into 96-well microplates and incubated for 2 hours at 37°C and then the liquid of each wells were removed without washing. The wells were incubated for 1 hour with 100 μl biotin-antibody at 37°C. After washing, HRP-avidin working solution was added to the wells and incubated for 1 hour at 37°C. Tetramethylbenzidine (TMB) substrate solutions were added to the wells and incubated for 30 minutes at room temperature in the dark. After addition of the stop solution, the absorbance was measured using a microplate reader at 450 nm. All samples and the standards were analyzed in duplicate and the average of the duplicates was used for analysis. Serum Rab35 concentrations were calculated using the kit-specific standard curves generated by CurveExpert 1.4 software.

### Transgenic mice expressing mutant (R1441C) LRRK2 or (G2019S) LRRK2

The transgene construct was prepared by inserting cDNA of HA-tagged mutant (R1441C) LRRK2 or (G2019S) LRRK2 into CMV enhancer/platelet-derived growth factor (PDGF)-β chain expression vector. Then, linearized transgene construct was purified and injected into male pronuclei of fertilized oocytes prepared from FVB/N mice, which were then implanted into pseudopregnant mice. Founder transgenic mice were mated with wild-type FVB/N mice and bred into stable transgenic lines. According to previous report, (R1441C) LRRK2 or (G2019S) LRRK2 transgenic mice at the age of 12-16 months exhibited the degeneration of SNpc dopaminergic neurons and parkinsonism phenotypes of motor dysfunction [14, 15].

### MPTP-treated and rotenone-treated mouse model of PD

All animals used for this study were approved by the Institutional Animal Care and Use Committee of Chang Gung University (No. 2013051501). The dose dependent experiments were conducted with three different doses of 1-methyl-4-phenyl-1,2,3,6-tetrahydropyridine (MPTP; Sigma-Aldrich) (20, 30, and 40 mg/kg) to determine the expression of Rab35 in MPTP-induced PD model. Mice were intraperitoneally (i.p.) injected with saline in the control group and with different dose of MPTP for 14 days. In rotenone treated group, mice were treated with rotenone (50 mg/kg/day, oral administration) for 14 days. At 14 days after saline, MPTP or rotenone administration, mice were sacrificed and brain tissues were excised for western blot analysis.

### Cell culture, transfection and immunofluorescent staining

The cDNA of Rab35 (GenBank ID: BT020024), wild-type (WT) alpha-synuclein (α-Syn) or A53T alpha-synuclein (A53T) was subcloned into a mammalian expression vector pcDNA3 (Invitrogen). SH-SY5Y cells were grown on coverslips and transfected with plasmid DNA using Lipofectamine 2000 (Invitrogen) according to the manufacturer's instructions. Two days after transfection, the transfected SH-SY5Y cells were fixed with 4% paraformaldehyde for 10 minutes and then permeabilized with 0.5% Triton X-100 in PBS. After washing three times, fixed cells blocked with 5% FBS and incubated with anti-Rab35 or anti-α-synuclein primary antibody. The cells were then stained with Alexa Fluor 488- or 594-conjugated secondary antibodies (Invitrogen) for 1 hour at room temperature. The fluorescence was visualized using a Leica DM6000 microscope equipped with Leica TCS SP5 confocal spectral scanning system.

### Preparation of exosome-depleted serum and conditioned medium (CM)

The depletion of exosomes from fetal bovine serum was performed according to methods described in the literature [20,43]. Following centrifugation at 100,000 × g for 16 hours at 4°C, the supernatant was collected and filtrated through a 0.2-μm filter and stored at 4°C until ready to use. For the conditioned medium, the growth medium was replaced with DMEM/F12 medium containing 2% exosome-depleted FBS. Two days after transfection, the CM was collected and centrifuged at 4000 × g for 10 min at 4°C to remove cell debris and dead cells. The supernatant was concentrated using 3 kDa cutoff Amicon Ultra filters (Millipore, Billerica, MA, USA).

### Western blot analysis

The substantia nigra (SN) and striatum (ST) tissues were collected from wild-type mice, MPTP-treated mice, rotenone-treated mice, SCA3 transgenic mice, (R1441C) LRRK2 or (G2019S) LRRK2 transgenic mice. Briefly, the SN or ST tissues were homogenized with CHAPS lysis buffer containing protease inhibitors (protease inhibitor cocktail, Sigma-Aldrich). A total of 30 μg proteins was separated by 12% SDS-polyacrylamide gel and transferred to PVDF membrane. The membranes were blocked in 5% low-fat milk solution in phosphate-buffered saline with 0.05% Triton X-100. Then, the membranes were hybridized with anti-Rab35 antibody followed by incubation with secondary antibody conjugated to horseradish peroxidase (Santa Cruz Biotechnology, Dallas, TX). Immunoreactive proteins were visualized by using an enhanced chemiluminescence kit (GE Biosciences). To confirm equal amount of protein sample loading, membranes were stripped and reblotted with monoclonal anti-actin antibody (Millipore). Gel bands were quantified by a densitometer (Molecular Dynamics Model 375A) and normalized to an actin control band. Anti-Rab35 polyclonal antibody and monoclonal anti-α-synuclein antiserum were purchased from ProteinTech, Inc (Chicago, IL). The antibody against actin (clone C4) was obtained from Millipore (Darmstadt, Germany).

### Statistical analysis

The GraphPad Prism v5 software (GraphPad System Inc., CA, USA) and SPSS v12 software (SPSS Inc. Illinois, USA) were used for statistical analysis. Data are expressed as mean ± standard error of the mean (SEM). Statistical significance among multiple experimental groups was determined by one-way ANOVA followed by Tukey test. Nonparametric Mann-Whitney U test (two-tailed) was used to determine the significant differences between two groups of data. Pearson correlation coefficient analysis was used to measure the correlation between two variables. Differences were considered statistically significant when the *P* value was less than 0.05. Receiver operating characteristic (ROC) curves were used to evaluate the efficacy of biomarkers at different cut-off points. The area under the curve (AUC) was calculated for biomarker, in order to assess the biomarkers on PD predicting. Linear regression analysis was used to evaluate the association of Rab35 with clinical parameters such as age at onset (AAO) or disease duration. The survival differences of PD patients in two groups were evaluated by the log-rank test.

## SUPPLEMENTARY MATERIALS





## ABBREVIATIONS

PD: Parkinson's disease
α-Syn: α-synuclein
Rab35: Ras-related protein 35
MPTP: 1-Methyl-4-phenyl-1,2,3,6-tetrahydropyridine
PSP: progressive supranuclear palsy
MSA: multiple system atrophy
