# Supplementary material for: Increased Rab35 expression is a potential biomarker and implicated in the pathogenesis of Parkinson's disease
Source: Oncotarget. 2016 Aug 5;7(34):54215–27. doi: 10.18632/oncotarget.11090 (PMC5342336; doi:10.18632/oncotarget.11090)
Supplement: Supplementary file 1 [file oncotarget-07-54215-s001.pdf]

Increased Rab35 expression is a potential biomarker and implicated in the pathogenesis of Parkinson's disease

Supplementary Material

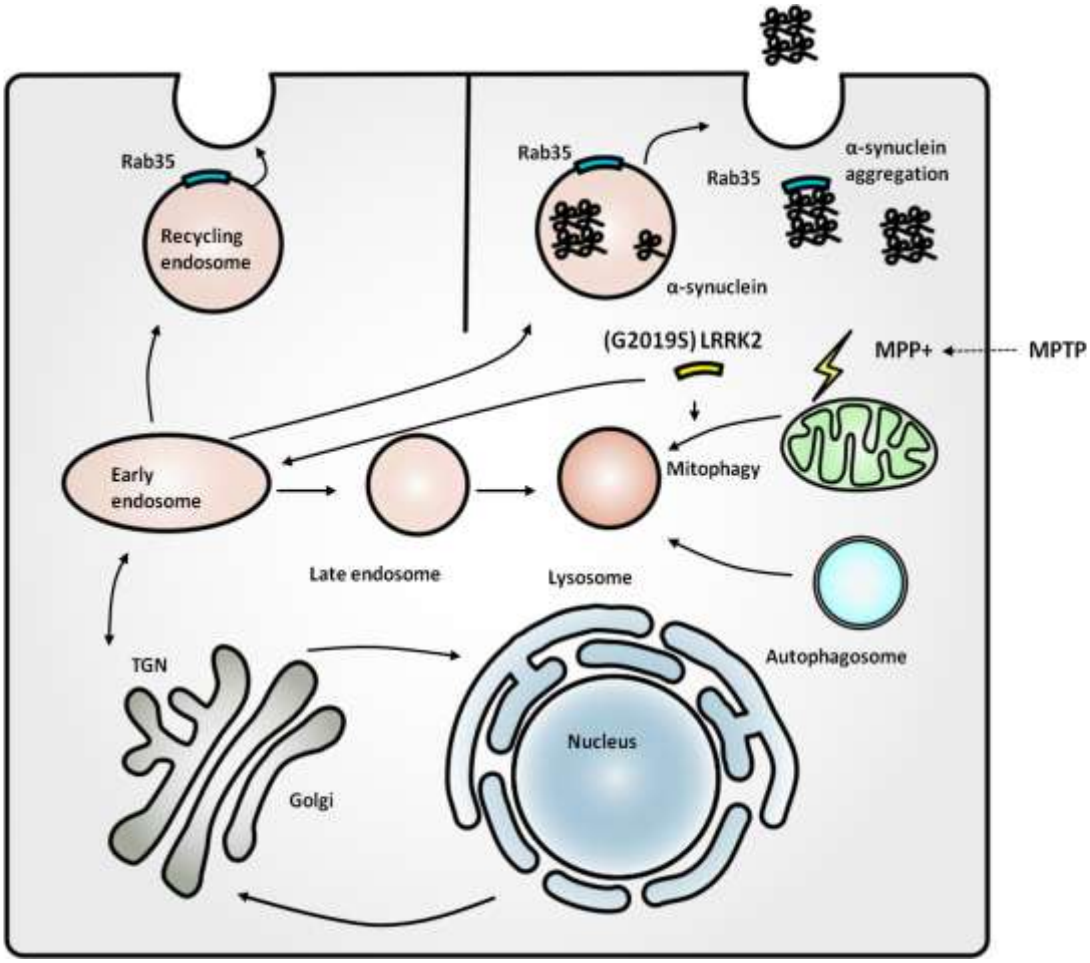

Supplemental Figure 1: Illustration of the possible mechanism underlying the role of Rab35 in pathogenesis of PD. Left panel: under a normal condition, Rab35 is localized in recycling endosome trafficking to the cell surface. Right panel: under the PD condition, the dysfunction of protein degradation results in the sequestration of abnormal proteins in Rab35 localized endosome. Overexpression of Rab35 promotes the aggregation of α-Syn and secretion of α-Syn to the intercellular space.

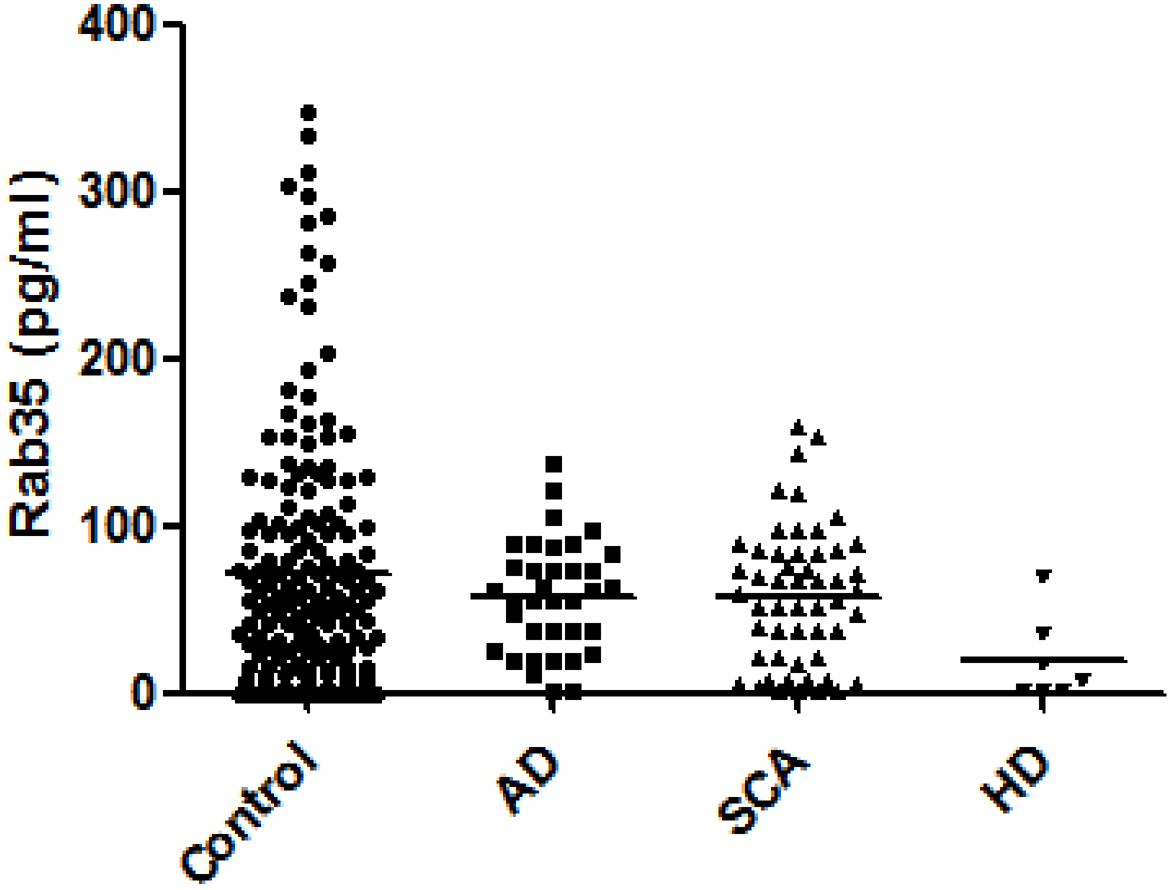

Supplemental Figure 2: The serum level of Rab35 is not significantly increased in patients with Alzheimer's disease (AD), spinocerebellar ataxia (SCA) or Huntington's disease (HD). The dot plot shows the serum level of Rab35 in healthy control subjects (n=177), AD patients (n=36), SCA patients (n=53) or HD patients (n=7).

Supplemental Table 1: Demographic data and Rab35 serum levels of patients and control subjects

Group	Controls	PSP	MSA	PD	PD	
AAO					AAO≦50	AAO>50
N	177	46	80	213	32	181
Gender (M / F)	99 / 88	25 / 21	35 / 45	107 / 106	17 / 15	90 / 91
Age (years)*	61.60 ± 0.94	61.98 ± 1.13	57.75 ± 0.9	62.12 ± 0.94	35.91 ± 1.54	66.75 ± 0.59
Serum Rab35 level (pg/ml)*	70.51 ± 5.72	71.22 ± 9.37	58.37 ± 8.22	131.30 ± 9.67	210.35 ± 28.29	117.32 ± 9.90

PSP, progressive supranuclear palsy; MSA, multiple system atrophy; PD, Parkinson's disease

* Mean ± SEM

Supplemental Table 2: Identification of serum proteins differentially expressed between NC and PD.

For Table S2, please see the attached Excel file
